# Supplementary material for: Pyruvate Dehydrogenase Kinase 1 inhibition mediated oxidative phosphorylation enhancement in cartilage promotes osteoarthritis progression
Source: BMC Musculoskelet Disord. 2023 Jul 20;24:597. doi: 10.1186/s12891-023-06585-6 (PMC10357736; doi:10.1186/s12891-023-06585-6)
Supplement: Supplementary file 1 — Additional file 1. [file 12891_2023_6585_MOESM1_ESM.zip › BP.Gsea.1653623667859/GOBP_LYMPH_VESSEL_MORPHOGENESIS.html]

Details for gene set GOBP\_LYMPH\_VESSEL\_MORPHOGENESIS[GSEA]

|  || Dataset | 55457\_collapsed\_to\_symbols.cls.cls #OA\_versus\_normal.cls.cls #OA\_versus\_normal\_repos |
| Phenotype | cls.cls#OA\_versus\_normal\_repos |
| Upregulated in class | normal |
| GeneSet | GOBP\_LYMPH\_VESSEL\_MORPHOGENESIS |
| Enrichment Score (ES) | -0.5598811 |
| Normalized Enrichment Score (NES) | -1.7216467 |
| Nominal p-value | 0.005226481 |
| FDR q-value | 0.122351564 |
| FWER p-Value | 1.0 |
Table: GSEA Results Summary

  

Fig 1: Enrichment plot: GOBP\_LYMPH\_VESSEL\_MORPHOGENESIS      
 Profile of the Running ES Score & Positions of GeneSet Members on the Rank Ordered List

  

| SYMBOL | TITLE | RANK IN GENE LIST | RANK METRIC SCORE | RUNNING ES | CORE ENRICHMENT || 1 | VEGFC | vascular endothelial growth factor C [Source:HGNC Symbol;Acc:HGNC:12682] | 2239 | 0.335 | -0.1210 | No |
| 2 | PROX1 | prospero homeobox 1 [Source:HGNC Symbol;Acc:HGNC:9459] | 4299 | 0.128 | -0.2606 | No |
| 3 | FOXC1 | forkhead box C1 [Source:HGNC Symbol;Acc:HGNC:3800] | 5227 | 0.054 | -0.3240 | No |
| 4 | PPP3CB | protein phosphatase 3 catalytic subunit beta [Source:HGNC Symbol;Acc:HGNC:9315] | 5311 | 0.046 | -0.3231 | No |
| 5 | PDPN | podoplanin [Source:HGNC Symbol;Acc:HGNC:29602] | 5594 | 0.024 | -0.3411 | No |
| 6 | SOX18 | SRY-box transcription factor 18 [Source:HGNC Symbol;Acc:HGNC:11194] | 6053 | -0.008 | -0.3754 | No |
| 7 | SVEP1 | "sushi, von Willebrand factor type A, EGF and pentraxin domain containing 1 [Source:HGNC Symbol;Acc:HGNC:15985]" | 8431 | -0.188 | -0.5302 | Yes |
| 8 | VASH1 | vasohibin 1 [Source:HGNC Symbol;Acc:HGNC:19964] | 8473 | -0.191 | -0.5032 | Yes |
| 9 | PKD1 | "polycystin 1, transient receptor potential channel interacting [Source:HGNC Symbol;Acc:HGNC:9008]" | 8755 | -0.213 | -0.4914 | Yes |
| 10 | LGALS8 | galectin 8 [Source:HGNC Symbol;Acc:HGNC:6569] | 9424 | -0.266 | -0.5012 | Yes |
| 11 | TIE1 | tyrosine kinase with immunoglobulin like and EGF like domains 1 [Source:HGNC Symbol;Acc:HGNC:11809] | 9548 | -0.278 | -0.4669 | Yes |
| 12 | EPHA2 | EPH receptor A2 [Source:HGNC Symbol;Acc:HGNC:3386] | 10219 | -0.343 | -0.4647 | Yes |
| 13 | ACVR2B | activin A receptor type 2B [Source:HGNC Symbol;Acc:HGNC:174] | 11011 | -0.431 | -0.4581 | Yes |
| 14 | BMPR2 | bone morphogenetic protein receptor type 2 [Source:HGNC Symbol;Acc:HGNC:1078] | 11174 | -0.454 | -0.3990 | Yes |
| 15 | PTPN14 | protein tyrosine phosphatase non-receptor type 14 [Source:HGNC Symbol;Acc:HGNC:9647] | 11797 | -0.548 | -0.3607 | Yes |
| 16 | FOXC2 | forkhead box C2 [Source:HGNC Symbol;Acc:HGNC:3801] | 12218 | -0.650 | -0.2906 | Yes |
| 17 | ACVRL1 | activin A receptor like type 1 [Source:HGNC Symbol;Acc:HGNC:175] | 12242 | -0.656 | -0.1888 | Yes |
| 18 | FLT4 | fms related receptor tyrosine kinase 4 [Source:HGNC Symbol;Acc:HGNC:3767] | 12396 | -0.705 | -0.0893 | Yes |
| 19 | VEGFA | vascular endothelial growth factor A [Source:HGNC Symbol;Acc:HGNC:12680] | 12629 | -0.813 | 0.0210 | Yes |
Table: GSEA details [plain text format]

  

Fig 2: GOBP\_LYMPH\_VESSEL\_MORPHOGENESIS      
 Blue-Pink O' Gram in the Space of the Analyzed GeneSet

  

Fig 3: GOBP\_LYMPH\_VESSEL\_MORPHOGENESIS: Random ES distribution      
 Gene set null distribution of ES for **GOBP\_LYMPH\_VESSEL\_MORPHOGENESIS**
